# Vemurafenib Induces Apoptosis via JNK Activation and AKT Inhibition in Hepatocellular Carcinoma

**DOI:** 10.7150/jca.130097

**Published:** 2026-05-18

**Authors:** Yu Rim Cho, Ke Huang, Zhibin Liu, Hyeonjin Kim, Chae Yeon Kim, Lei Ma, Nangwon Yee, Chae Rim Kim, Geun Hye Park, Jiaxi Zhang, Zae Young Ryoo, Sung Hyun Kim, Myoung Ok Kim

**Affiliations:** 1 Department of Animal Science and Biotechnology, Research Institute for Innovative Animal Science, Kyungpook National University, Sangju, Gyeongsangbuk-do, Republic of Korea.; 2 Institute of Basic Science Conversions, Kyungpook National University, Daegu, Republic of Korea.; 3 Translational Medical Center, Huaihe Hospital, Henan University, Kaifeng, Henan, 475000, PR China.; 4 School of Life Sciences, BK21 FOUR KNU Creative BioResearch Group, Kyungpook National University, Daegu, Republic of Korea.; 5 Department of Bio Pharmaceutical Materials, Korea Polytechnic College, Daegu, Republic of Korea.

**Keywords:** hepatocellular carcinoma, vemurafenib, JNK, AKT, apoptosis

## Abstract

**Background:**

Hepatocellular carcinoma (HCC) is the most common form of primary liver cancer and remains one of the leading contributors to cancer-related mortality worldwide, primarily due to the disease's therapeutic resistance and marked tumor heterogeneity. Among the molecular mechanisms implicated in HCC progression, the dysregulation of the JNK and PI3K/AKT signaling pathways plays a critical role in tumor initiation, cell proliferation, and cell survival. Vemurafenib, a clinically approved inhibitor of mutant BRAF kinase, has shown robust antitumor activity across multiple malignancies. However, its therapeutic utility in HCC remains largely unexplored.

**Methods:**

We investigated the anticancer effects of vemurafenib in two human HCC cell lines.

**Results:**

Vemurafenib treatments led to significant declines in cell viability and colony formation, accompanied by marked reductions in the migratory and invasive behaviors of HCC cells. Mechanistically, vemurafenib enhanced JNK pathway activation while suppressing AKT phosphorylation.

**Conclusion:**

Vemurafenib markedly inhibited HCC cell proliferation and metastasis, which was accompanied by a pronounced induction of apoptosis and G0/G1 phase cell-cycle arrest. At the signaling level, these cellular responses were linked to enhanced JNK activation and the suppression of AKT phosphorylation, suggesting that vemurafenib may serve as a potential therapeutic agent for HCC.

## Introduction

Hepatocellular carcinoma (HCC) accounts for approximately 90% of all primary liver malignancies 1, 2. It manifests as a spectrum of systemic clinical symptoms, including fatigue, abdominal pain, abdominal distension, weight loss, jaundice, anorexia, and nausea 3, 4. HCC is a leading cause of cancer-related deaths, consistently ranking among the top five in global cancer mortality 5, 6. Currently, therapeutic options for HCC include surgical resection, radiotherapy, liver transplantation, transarterial chemoembolization, and systemic chemotherapy 7-9. Although surgical and locoregional interventions have undergone substantial improvements, patients with advanced-stage HCC continue to face dismal prognoses, which is largely attributable to the disease's therapeutic resistance and its marked molecular and cellular heterogeneity 10, 11.

To overcome these limitations, recent studies have increasingly explored novel therapeutic approaches aimed at modulating the intracellular signaling pathways that drive HCC progression. Among these, the MAPK/JNK and PI3K/AKT pathways, regulators of cell survival, proliferation, and apoptosis, have emerged as promising targets. Activation of the JNK signaling axis has been shown to trigger apoptotic cell death in multiple cancer types 12, 13, whereas the PI3K/AKT cascade predominantly promotes cell survival and suppresses apoptotic processes 14-19. Consequently, the concurrent activation of JNK signaling and inhibition of AKT activity could rationally be a strategy in anti-HCC therapy. However, the biological role of JNK in hepatocarcinogenesis is multifaceted. As summarized in a comprehensive review 20, persistent JNK1 activation has been implicated in the stimulation of cellular proliferation and alteration of metabolic programming, promoting tumor initiation and progression, whereas acute or stress-induced JNK activation can elicit pro-apoptotic and tumor-suppressive responses. These context-dependent roles show that, if JNK is to be used as a therapeutic target, precise modulation will be key, yet they also highlight the potential of therapeutic agents capable of selectively regulating this pathway. For instance, it has been reported that the PI3K/AKT/mTOR pathway is suppressed by (+)-ABX, leading to cell cycle arrest at the S and G2/M phases, apoptosis induction, and autophagy activation in non-small cell lung cancer (NSCLC) cells 21. Similarly, sunitinib has been shown to increase TRAIL-mediated apoptosis through enhanced JNK activation, the downregulation of XIAP, and the promotion of apoptotic signaling in colon cancer 22.

**Figure 1:**
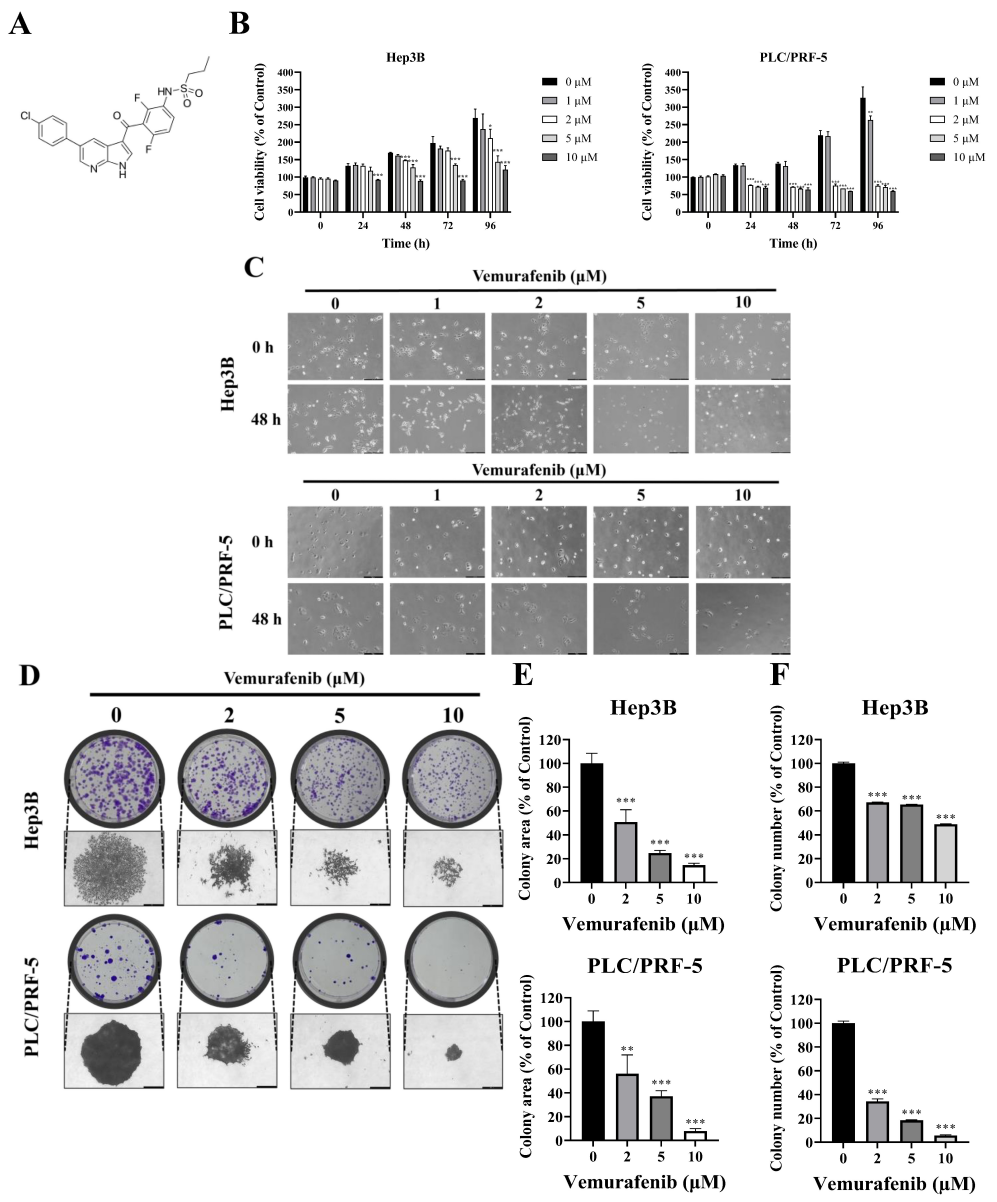
Vemurafenib inhibits the proliferation of HCC cells. (A) The chemical structure of vemurafenib. (B) HCC cells were exposed to 0, 1, 2, 5, and 10 μM of vemurafenib for 0, 24, 48, 72, and 96 h, and cell viability was assessed using the CCK-8 assay. (C) Representative phase-contrast microscope images (200× magnification) showing morphological alterations of HCC cells following treatment with the indicated concentrations of vemurafenib for 0 and 48 h. (D-F) The colony-forming abilities of Hep3B and PLC/PRF-5 cells after vemurafenib treatments: (D) representative images of colony plates (top) and individual colonies (bottom; 200× magnification), and (E) the total areas and (F) the numbers of colonies, as percentages relative to the control. In panels B, E, and F, significant differences between the treatment and control (0 μΜ) groups are denoted using asterisks: *, *p* < 0.05; **, *p* < 0.01; and ***, *p* < 0.001.

Vemurafenib (**Figure 1A**), a selective inhibitor of mutant BRAF kinase, was approved by the U.S. Food and Drug Administration (FDA) in 2011 for the treatment of melanoma 23, 24. Beyond its established clinical use, vemurafenib has demonstrated anticancer activity across a wide range of malignancies. During preclinical investigations using BRAF V600E-mutant colorectal cancer models, vemurafenib markedly attenuated tumor growth by disrupting MAPK pathway signaling 25. In NSCLC, vemurafenib exhibited clinical benefits in tumors harboring both BRAF V600E and non-V600 mutations, suggesting therapeutic potential beyond classical BRAF mutation cancer types 26. Importantly, the HCC cell lines used in this study (Hep3B and PLC/PRF-5) do not harbor canonical BRAF V600 mutations and were selected as commonly used HCC models to examine the effects of vemurafenib on phenotypic responses and intracellular signaling pathways relevant to HCC progression, including MAPK-, AKT-, and JNK-associated regulatory networks. Because the present study did not directly assess BRAF mutational dependency, the observed effects cannot be concluded to be either BRAF-dependent or BRAF-independent, and this question warrants further investigation. Notably, FDA-approved molecular therapy agents for HCC, including sorafenib and regorafenib, exert their anticancer effects via the inhibition of MAPK pathway components 27, 28. Given this mechanistic overlap, it is plausible that vemurafenib would also exhibit therapeutic effects against HCC, yet its anti-HCC potential remains insufficiently characterized. Therefore, the present study aimed to evaluate the anti-HCC effects of vemurafenib and elucidate the molecular mechanisms underlying its action.

## Materials and Methods

### Reagents and antibodies

Vemurafenib (PLX4032, RG7204; > 98% purity; Aladdin, Shanghai, China) was prepared as a stock solution in dimethyl sulfoxide. The final concentration of DMSO in all treatments and corresponding vehicle control groups was maintained below 0.1%. Primary antibodies, including Bcl-2 (sc-7382), E-cadherin (sc-21791), N-cadherin (sc-59987), p27(sc-56338), and β-actin (sc-47778), were sourced from Santa Cruz Biotechnology (Dallas, TX, USA), and antibodies against Bax (2772S), Cyclin D1 (2922S), CDK4 (12790S), cytochrome c (4272S), p-JNK (9251S), JNK (9252S), p-AKT (9271S), and AKT (9272S) were purchased from Cell Signaling Technology (Danvers, MA, USA).

### Cell culture

Two human hepatocellular carcinoma (HCC) cell lines, Hep3B and PLC/PRF-5, were obtained from the Korean Cell Line Bank (Seoul, Korea) and used in all experiments. The Hep3B cells were maintained in Dulbecco's modified Eagle's medium (Gibco™, Grand Island, NY, USA), and PLC/PRF-5 cells in Roswell Park Memorial Institute 1640 medium (Gibco™, Grand Island, NY, USA), each supplemented with fetal bovine serum (FBS; GenDEPOT, Katy, TX, USA) at 10% and penicillin-streptomycin (Gibco™, NY, USA) at 1%. Cells were cultured at 37°C in a humidified atmosphere containing 5% CO₂.

### Cell viability assays

Cell viability was assessed using the Cell Counting Kit-8 (CCK-8; Dojindo, Kumamoto, Japan) assay. For both cell types, cells were seeded into 96-well plates at 1 × 10³ cells/well and incubated for 12 h to allow cell adhesion. The medium was replaced with fresh medium containing 0, 1, 2, 5, or 10 μM of vemurafenib, and viability was measured at 0, 24, 48, 72, and 96 h by adding 10 μL of CCK-8 per well and incubating the mixture for 1 h at 37°C. The optical ensity at 450 nm was then recorded using a microplate reader (BioTek Instruments, Winooski, VT, USA).

### Morphological analysis

For both cell types, cells were seeded at 5 × 10⁴ cells/well into 6-well plates and allowed to adhere for 24 h. The cells were then exposed to 0, 1, 2, 5, or 10 μM vemurafenib. Cellular morphology, focusing on cell shape, adherence, and confluence, was examined at 0 and 48 h post-treatment under a phase-contrast inverted microscope.

### Colony formation assays

For both cell types, cells were seeded at 1 × 10^3^ cells/well into 6-well plates and treated with 0, 2, 5, or 10 μM vemurafenib. Colonies were allowed to form for 14 d under standard conditions, fixed in methanol, stained with 0.2% crystal violet, and then photographed under an inverted microscope (Leica Microsystems, Wetzlar, Germany), with quantification using ImageJ (v1.53e; National Institutes of Health, Bethesda, MD, USA).

### Wound-healing assays

Cells were grown in 6-well plates to near-complete confluence (~100%). A uniform scratch was created using a sterile 200 μL pipette tip, and the wells were gently washed with phosphate-buffered saline (PBS). The medium was then replaced with fresh medium containing 0, 2, 5, or 10μM of vemurafenib. The wounds were imaged at 0 h and 30 h using a phase-contrast microscope, and migration distance was quantified with ImageJ (v1.53e; National Institutes of Health) to assess closure.

### Invasion assays

Cell invasion was evaluated using 24-well Transwell chambers equipped with 8.0 μm pore polycarbonate membranes (Corning, NY, USA) pre-coated with Matrigel (Corning Costar, Lowell, MA, USA). Cells were resuspended in 200 μL serum-free medium containing 0, 2, 5, or 10 μM of vemurafenib and seeded into the upper chamber at 1 × 10⁴ cells/well. The lower chamber contained 600 μL of complete medium with FBS at 10% as a chemoattractant. After a 48-hour incubation at 37°C in 5% CO₂, non-invading cells were gently removed from the upper surface with a cotton swab. Invading cells on the underside were fixed in methanol for 20 min, stained with 0.2% crystal violet, and then imaged and quantified using ImageJ (v1.53e; National Institutes of Health).

### Western blotting

For both cell types, cells were seeded at 1 × 10^6^ cells/dish into 10 cm dishes and treated with 0, 2, 5, or 10 μM vemurafenib for 48 h at 37°C. The cells were then lysed in EDTA-free RIPA II buffer (1×) supplemented with a protease inhibitor cocktail (100×) (GenDEPOT, Katy, TX, USA). Protein concentrations were determined using the BCA assay (Thermo Fisher Scientific, Waltham, MA, USA) on a NanoDrop^TM^ 2000. Equal amounts of protein (30 μg) were separated by SDS-PAGE and transferred to polyvinylidene difluoride membranes (0.22 μm; Merck Millipore, Darmstadt, Germany). The membranes were then blocked with a 5% nonfat dry milk and 0.1% Tween-20 in tris-buffered saline, incubated with the primary antibody overnight at 4°C, washed, and incubated with the appropriate HRP-conjugated secondary antibody for 1 h at room temperature. Bands were visualized using enhanced chemiluminescence (Bio-Rad, Hercules, CA, USA) and imaged in an ImageQuant LAS 500 system (Cytiva, Marlborough, MA, USA).

### Apoptosis analysis

For both cell types, cells were seeded into 6-well plates at 1 × 10⁵ cells/well, allowed to adhere overnight at 37 °C in 5% CO₂, and then exposed to 0, 2, 5, or 10 μM vemurafenib for 48 h. After vemurafenib exposure, both adherent and floating cells were harvested, washed twice with ice-cold PBS, resuspended in 1× annexin-binding buffer, and stained with annexin V-FITC and propidium iodide (Invitrogen, Thermo Fisher Scientific, Waltham, MA, USA) for 15 min at room temperature in the dark. Samples were analyzed on an FACSVerse flow cytometer (Thermo Fisher Scientific), and early- and late-stage apoptosis was quantified using appropriate gating parameters. Gating was performed based on unstained and single-stained controls to define viable (Annexin V^-^/PI^-^), early apoptotic (Annexin V^+^/PI^-^), and late apoptotic/necrotic (Annexin V^+^/PI^+^) cell populations.

### Cell-cycle analysis

For both cell types, cells were seeded into 6-well plates at 1 × 10^5^ cells/well, incubated overnight, and then treated with 0, 2, 5, or 10 μM vemurafenib for 48 h. The cells were detached with trypsin, pelleted (1000 rpm for 5 min), washed twice with ice-cold PBS, and fixed in 70% ethanol at 4°C. After washing, the cells were incubated with 0.5 mL of FxCycle^TM^ PI/RNase Staining Solution (Thermo Fisher Scientific) for 15 min at room temperature in the dark, and cellular DNA content was analyzed on a FACS Verse flow cytometry (Thermo Fisher Scientific) to assess the cell-cycle distribution.

### Database analysis

Data on JNK expression in HCC and adjacent non-tumorous liver tissues were obtained from the Gene Expression Omnibus (GEO) dataset GSE112790 and processed in R software (v4.1.0). The expression of AKT in and overall survival (OS) of liver hepatocellular carcinoma (LIHC) patients, stratified by high and low AKT expression, were analyzed using GEPIA (http://gepia.cancer-pku.cn/).

### Statistical analysis

All experiments used at least three independent biological replicates, and data are presented as means ± standard deviation (SD). Group comparisons were performed by one-way analysis of variance (ANOVA) followed by the appropriate post hoc multiple-comparison test, with *p* < 0.05 accepted as indicating statistical significance. Analyses and graphs were generated using GraphPad Prism version 9.0 (GraphPad Software, San Diego, CA, USA).

## Results

### Vemurafenib exerts antiproliferative activity against HCC cells

Cell viability was assessed at 0, 24, 48, 72, and 96 h after treatments with vemurafenib using the CCK-8 assay. The treatments led to marked dose- and time-dependent reductions in cell viability in both HCC cell lines (**Figure 1B**). To further examine vemurafenib's antiproliferative effects, Hep3B and PLC/PRF-5 cells were treated with 0, 1, 2, 5, and 10 μM vemurafenib and examined under a microscope. As the concentration increased, cell densities progressively declined (**Figure 1C**). Given its weak, non-significant inhibitory effect, the 1 μM concentration was excluded from subsequent experiments. Consistent with the cell viability and density result, colony formation assays demonstrated significant, dose-dependent reductions in clonogenic growth following vemurafenib exposure (**Figure 1D-F**). Collectively, these results indicate that vemurafenib exhibits potent antiproliferative activity against HCC cells, attenuating both short-term cell viability and long-term proliferative capacity.

### Vemurafenib inhibits the migration and invasion of HCC cells by modulating EMT-related pathways

**Figure 2:**
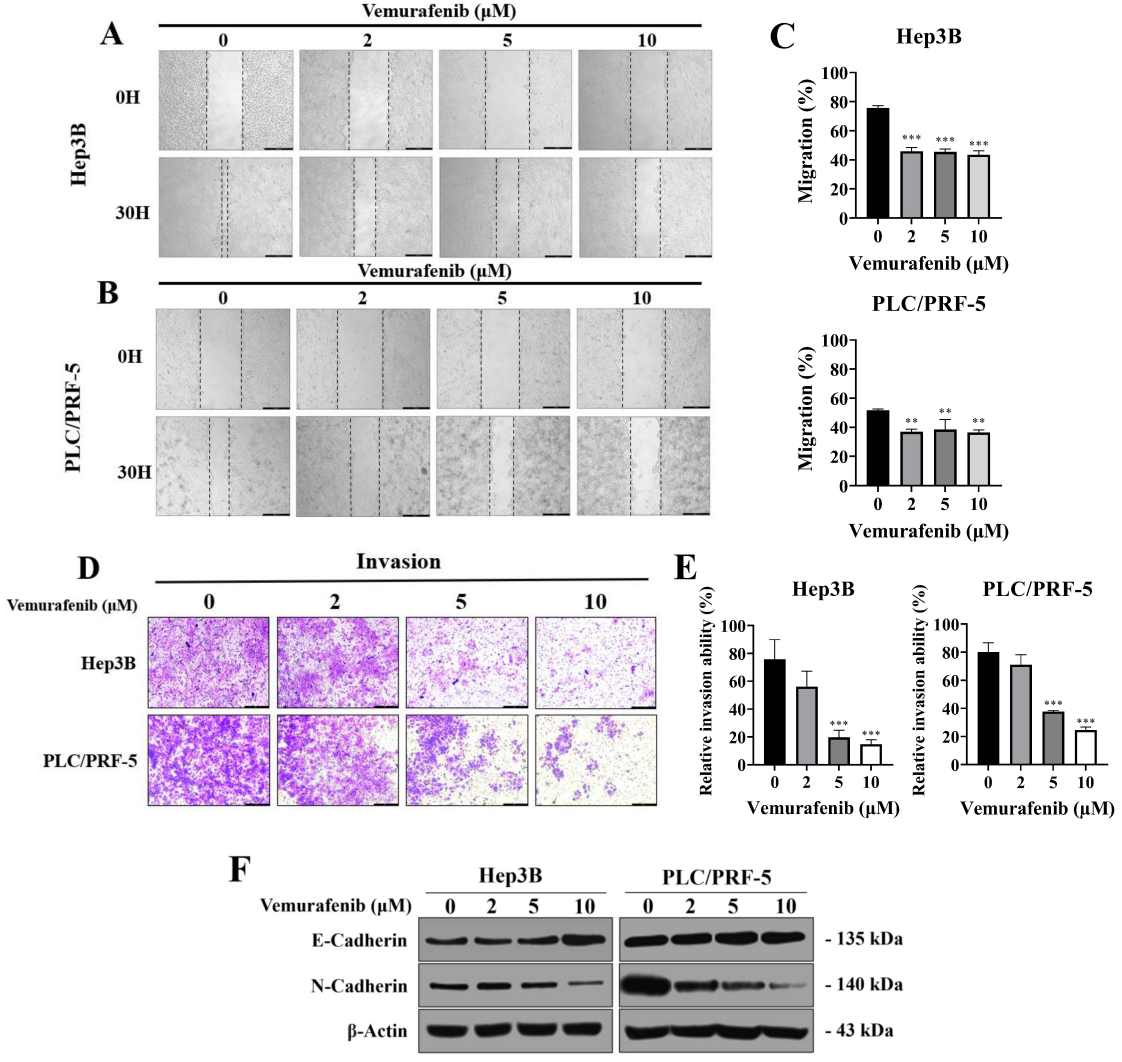
Vemurafenib suppresses the migratory and invasive abilities of HCC cells. (A-C) Representative wound-healing assay images, taken 0 and 30 h after scratching, showing the migratory behaviors of (A) Hep3B and (B) PLC/PRF-5 cells treated with 0, 2, 5, and 10 μM of vemurafenib, along with (C) graphs showing the migration rates of both HCC cell types. (D) Representative images showing Transwell invasion assay results for HCC cells treated with vemurafenib for 48 h, and (E) the invasive capacities of HCC cells, as percentages relative to the control (0 μM). (F) A western blot membrane image showing changes in E-cadherin and N-cadherin protein levels in both cell lines after 48 h of vemurafenib exposure at the indicated concentrations. In panels C and E, significant differences between the treatment and control (0 μM) groups are denoted using asterisks: *, *p* < 0.05; **, *p* < 0.01; and ***, *p* < 0.001.

To assess the impact of vemurafenib on migratory and invasive behaviors, HCC cells were subjected to wound-healing and Transwell invasion assays. In the wound-healing assay, vemurafenib exposure at 0, 2, 5, and 10 μM for 30 h markedly impeded the migration of both Hep3B and PLC/PRF-5 cells, reducing wound closure relative to the control group at all concentrations (**Figure 2A**-**C**). Similarly, the Transwell assay revealed that vemurafenib caused dose-dependent reductions in invasive capability in both cell lines (**Figure 2D**, **E**). To determine whether these effects were associated with epithelial mesenchymal transition (EMT) modulation, western blotting was performed to examine the expression of two major EMT markers. Treatment with vemurafenib led to an increase in E-cadherin expression and a concurrent decrease in N-cadherin expression in both HCC cell lines, suggesting inhibition of EMT (**Figure 2F**). Collectively, these findings demonstrate that vemurafenib suppresses HCC cell migration and invasion by modulating EMT-related pathways.

### Vemurafenib induces apoptosis and G0/G1 cell-cycle arrest in HCC cells

**Figure 3:**
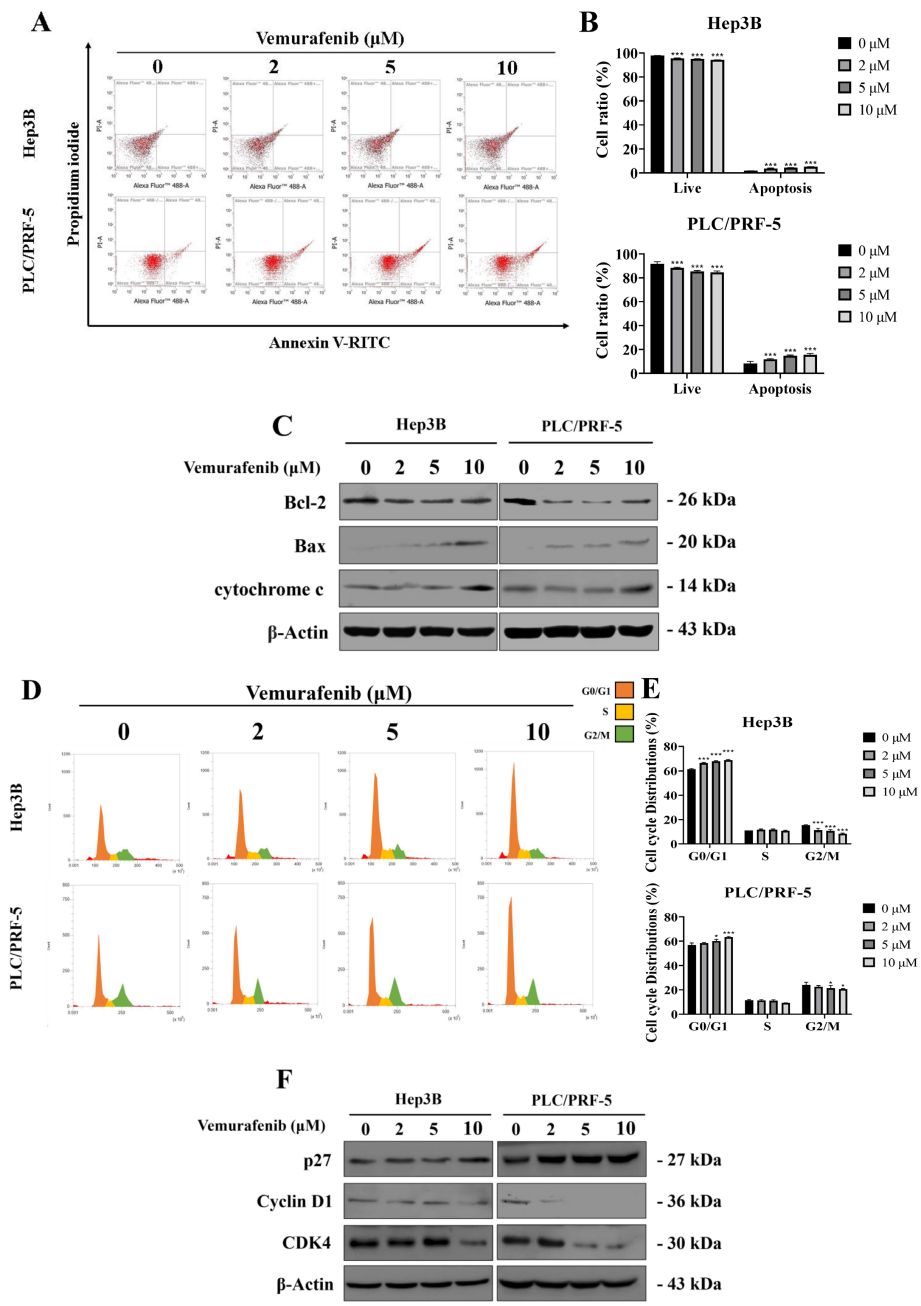
Vemurafenib triggers apoptosis and cell-cycle arrest in HCC cells. (A, B) Flow cytometric analysis results showing changes in apoptotic cell populations for Hep3B and PLC/PRF-5 cells following vemurafenib treatments, including (A) representative inverted-microscopy images and (B) bar graphs. (C) A representative western blot membrane showing apoptosis-related protein (Bax, Bcl-2, and cytochrome c) expression in HCC cells treated with vemurafenib. (D, E) Flow cytometric analysis results showing cell-cycle distributions in HCC cells after vemurafenib exposure, including (D) representative inverted-microscopy images and (E) bar graphs of the percentage of cells in each cell-cycle phase. (F) A representative western blot membrane showing cell-cycle regulatory protein (p27, Cyclin D1, and CDK4) expression in vemurafenib-treated HCC cells. In panels B and E, significant differences between the treatment and control (0 μM) groups are denoted using asterisks: *, *p* < 0.05; **, *p* < 0.01; and ***, *p* < 0.001.

To further elucidate the molecular mechanisms underlying vemurafenib's effects on HCC cells proliferation, apoptosis, and cell-cycle distributions were analyzed by flow cytometry following 48 h of treatment. Vemurafenib treatments markedly decreased viable cell proportions while increasing apoptotic populations in both Hep3B and PLC/PRF-5 cells (**Figure 3A**, **B**). Consistent with these observations, a western blot analysis demonstrated the elevated expression of pro-apoptotic proteins, including Bax and cytochrome c, along with reduced anti-apoptotic protein Bcl-2 levels, suggesting activation of the intrinsic mitochondrial apoptotic pathway (**Figure 3C**).

The cell-cycle analysis revealed a dose-dependent accumulation of cells in the G0/G1 phase, accompanied by a corresponding decrease in the G2/M population (**Figure 3D**, **E**). Furthermore, western blotting showed that the vemurafenib treatments markedly downregulated G0/G1 regulatory proteins, such as Cyclin D1 and CDK4, while upregulating the cyclin-dependent kinase inhibitor p27, thereby reinforcing G0/G1 phase arrest (**Figure 3F**). Taken together, these findings suggest that vemurafenib inhibits HCC cell proliferation by concurrently inducing apoptosis and enhancing G0/G1 cell-cycle arrest via modulation of apoptotic and cell-cycle regulatory pathways.

### Vemurafenib regulates JNK and AKT signaling in HCC cells

**Figure 4:**
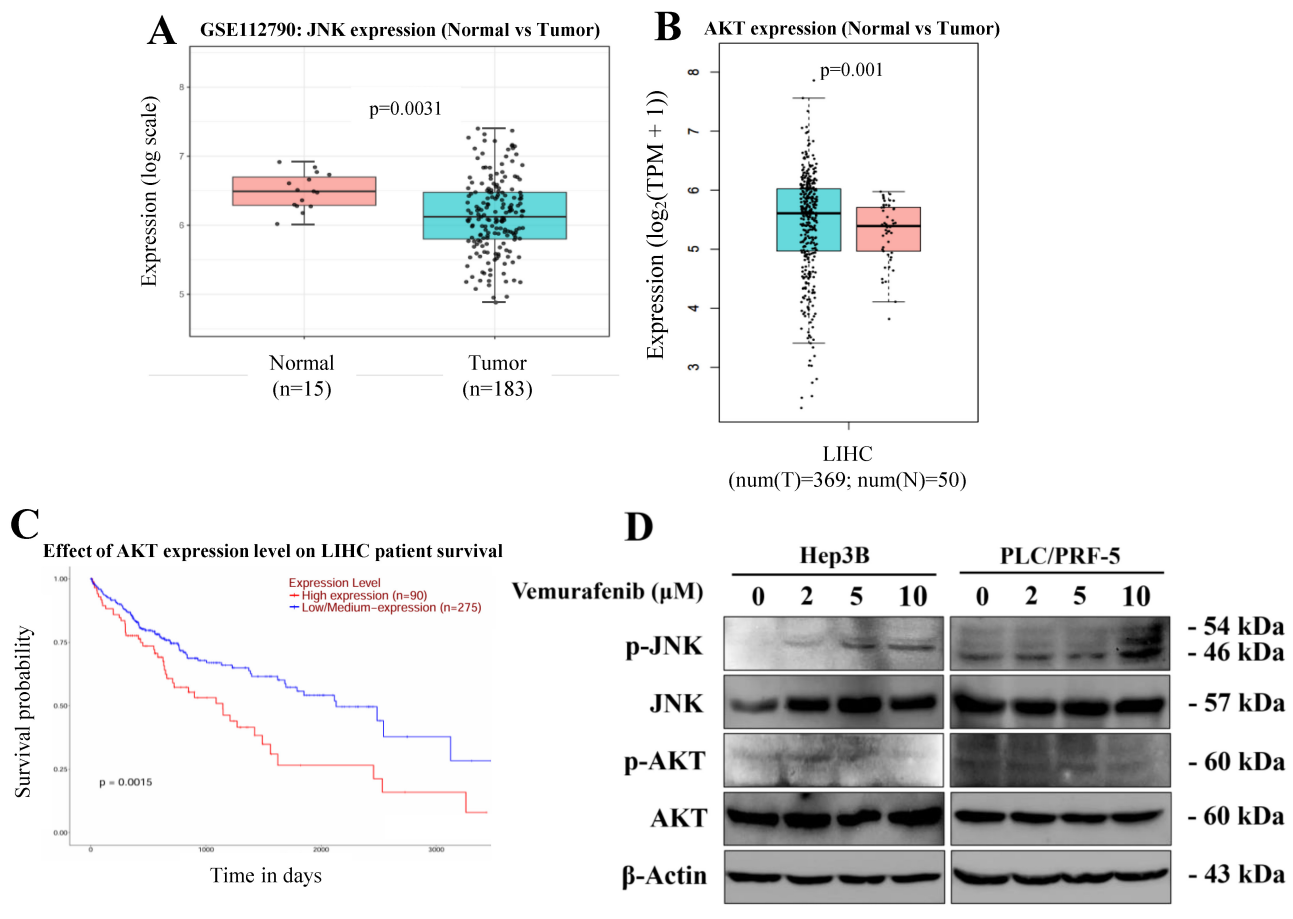
Vemurafenib regulates the JNK and AKT signaling pathways in HCC cells. (A, B) Box plots comparing (A) JNK and (B) AKT expression between normal liver and HCC tumor tissues. (C) A Kaplan-Meier survival curve illustrating the overall survival of LIHC patients stratified by AKT expression levels. (D) A representative western blot membrane image showing the expression of proteins associated with the JNK and AKT signaling pathways, including total JNK, phosphorylated JNK (p-JNK), total AKT, and phosphorylated AKT (p-AKT), in Hep3B and PLC/PRF-5 cells treated with vemurafenib.

Given vemurafenib's modulatory effects on apoptosis and the cell cycle in HCC cells, we explored the involvement of the JNK and AKT signaling pathways, negative and positive regulators of cell survival, respectively, combining a bioinformatic analysis based on publicly available data detailing JNK and AKT expression in HCC patients with an experimental assessment of vemurafenib's effects on these regulatory pathways. Our bioinformatic analysis, based on a GEO dataset, showed that, when compared to that of adjacent normal liver samples, JNK expression was markedly downregulated in HCC tumor tissues (**Figure 4A**). In contrast, the GEPIA 29 analysis revealed that AKT expression was higher in HCC tumor tissue than in normal liver tissues (**Figure 4B**). Moreover, a Kaplan Meier survival analysis indicated that patients with higher AKT expression exhibited significantly poorer overall survival than those with lower AKT levels (**Figure 4C**). To experimentally verify these findings at the protein level, western blotting was performed using Hep3B and PLC/PRF-5 cells treated with vemurafenib. Vemurafenib treatments induced a dose-dependent increase in phosphorylated JNK (p-JNK) and a concomitant decrease in phosphorylated AKT (p-AKT) (**Figure 4D**). Together, these results suggest that vemurafenib-induced pro-apoptotic effects in HCC cells are associated with enhanced JNK activation and suppressed AKT-mediated survival signaling.

## Discussion

In this study, we demonstrated that vemurafenib exhibits potent anticancer activity against HCC. Our results indicate that vemurafenib impairs not only the cellular proliferation and clonogenic potential of HCC cells but also their migratory and invasive capabilities, with the anti-migratory effect primarily supported by Transwell invasion assays and EMT marker modulation. Vemurafenib's anticancer properties were then shown to be driven by its ability to induce apoptotic cell death and G0/G1 phase arrest, and at the molecular level, these effects were linked to inhibition of the AKT signaling cascade and activation of the JNK pathway. Collectively, these findings suggest that vemurafenib, in conjunction with existing therapeutic modalities, may serve as a promising molecular candidate for HCC management.

**Figure 5:**
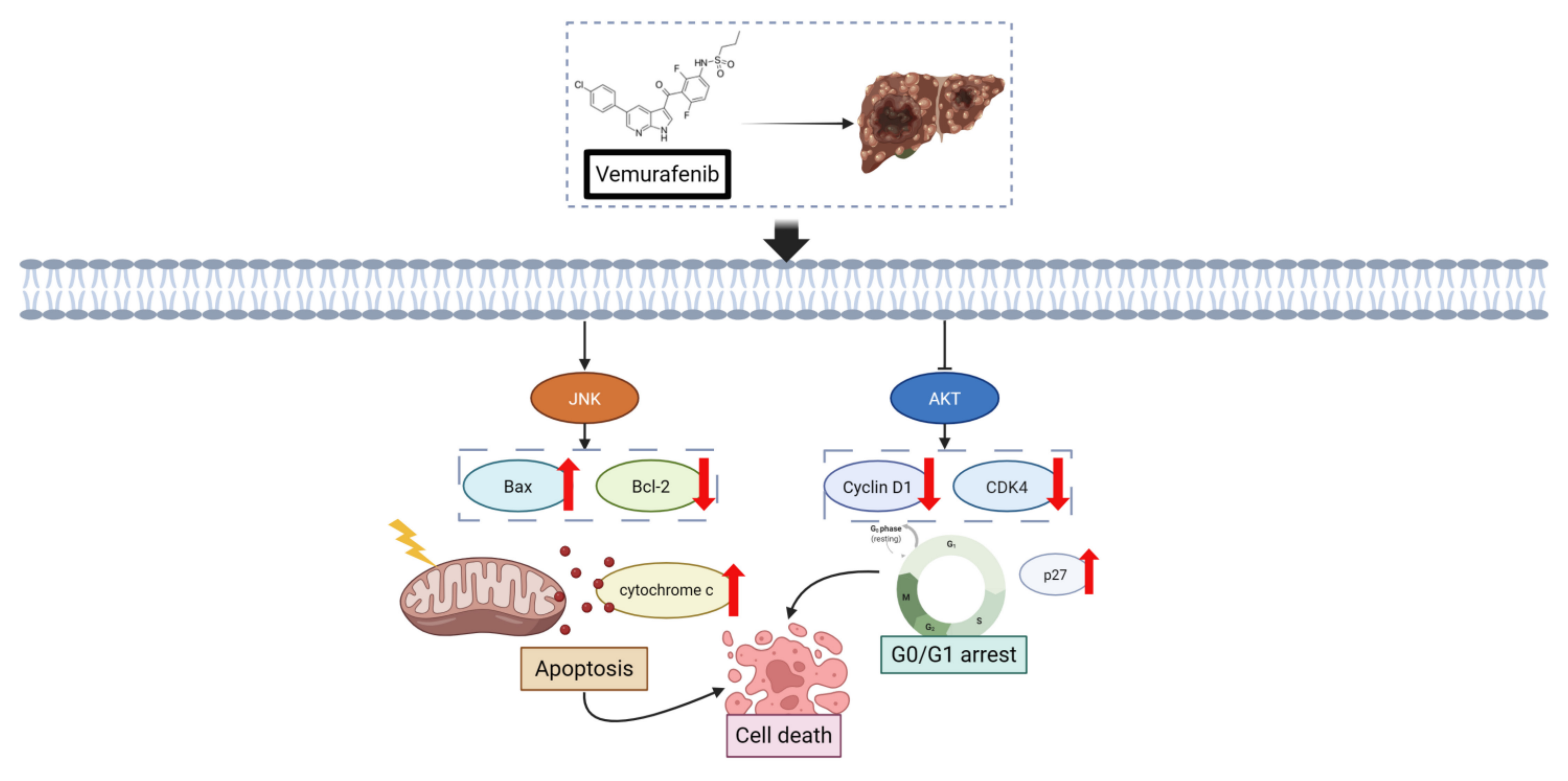
Schematic illustration of the proposed cellular responses to vemurafenib in HCC cells. Vemurafenib induces apoptosis through JNK activation and inhibits cell-cycle progression via AKT suppression, ultimately reducing HCC cell viability (Arrowheads indicate activation or upregulation, whereas blunt-ended lines indicate inhibition or suppression).

**Figure 5** shows a schematic illustration depicting the dual regulatory influence of vemurafenib on HCC cells based on this study's experimental evidence. Specifically, vemurafenib exposure enhances JNK signaling, leading to the upregulation of the pro-apoptotic effector Bax, suppression of the anti-apoptotic factor Bcl-2, and release of cytochrome c from mitochondria, thereby triggering the intrinsic apoptotic cascade. Concurrently, vemurafenib suppresses AKT signaling, resulting in the downregulation of Cyclin D1 and CDK4 and upregulation of the cell-cycle inhibitor p27. These alterations collectively culminate in G0/G1 cell-cycle arrest, thereby constraining the proliferative capacity of HCC cells.

Vemurafenib treatment also altered the cell-cycle landscape, increasing the G0/G1 fraction and decreasing the G2/M population. These observations align with the findings of prior HCC, colorectal, and breast cancer research, which also documented G0/G1 enrichment and G2/M depletion concurrent with apoptosis induction 30-32. Collectively, our findings, along with this previous research, strongly indicate that the antiproliferative activity of vemurafenib is executed through modulation of cell-cycle progression. Another compound, metformin, has also been demonstrated to inhibit HCC growth by inducing G0/G1 arrest through upregulation of p21 and p27 and downregulation of Cyclin D1, both in vitro and in vivo 30. Likewise, furanodienone has been shown to promote G0/G1 arrest and apoptosis in colorectal carcinoma cells via a ROS/MAPK-dependent caspase cascade 31, whereas euphol induces G1-phase arrest in breast carcinoma cells by modulating the expression of Cyclin D1, p21, and p27 32. Thus, our study adds to a body of research demonstrating the pivotal role of G0/G1 arrest and cell-cycle regulation as central mechanisms underlying the antiproliferative effects of diverse anticancer agents.

In prior research, vemurafenib has demonstrated anticancer efficacy in thyroid carcinoma cells by targeting the PI3K/AKT pathway, resulting in apoptosis and cell-cycle arrest 33, and vemurafenib's suppressive effects have been shown to be mediated by AKT signaling attenuation in breast carcinoma cells 34. Moreover, additional research indicates that PI3K/AKT pathway inhibition triggers apoptosis in HCC cells 35, while persistent AKT activation is associated with apoptosis suppression 36, 37. Conversely, in cutaneous squamous cell carcinoma, vemurafenib has been reported to inhibit JNK signaling, diminishing apoptotic activity 38. Previous studies using Hep3B cells have shown that natural compounds, such as iso-alantolactone and coptisine, promote apoptotic cell death via JNK pathway activation 39, 40. In hepatocellular carcinoma, JNK signaling has been more frequently linked to pro-apoptotic responses, particularly under conditions of acute or stress-induced activation. Previous studies using Hep3B cells have shown that natural compounds such as isoalantolactone and coptisine induce apoptotic cell death via activation of the JNK pathway 39, 40. In line with these findings, our results demonstrate that vemurafenib treatment induces JNK phosphorylation concomitant with mitochondrial apoptotic events, suggesting that JNK activation in this context represents an acute, pro-apoptotic stress response rather than a sustained oncogenic signal. Although upstream mechanisms were not directly examined, oxidative stress has been implicated as a common upstream trigger of stress-induced JNK activation in hepatocellular carcinoma, raising the possibility that ROS-mediated signaling may contribute to vemurafenib-induced JNK activation. Further studies will be required to elucidate the precise upstream regulators involved.

Since this investigation was performed exclusively in vitro, additional studies are needed to validate whether comparable effects occur in vivo. In addition, because this study was not designed to directly test BRAF dependency, it remains unclear whether the observed anticancer effects of vemurafenib in HCC cells are mediated through BRAF-dependent or BRAF-independent mechanisms. Before dissecting signaling mechanisms, we also analyzed a public transcriptomic database and observed that JNK expression was markedly reduced in HCC tissues compared to normal liver tissues, and vemurafenib treatment robustly activated JNK signaling in HCC cells. Similarly, although AKT transcript levels were significantly elevated in tumor tissue, vemurafenib potently inhibited AKT phosphorylation in HCC cells. Taken together, these findings support the therapeutic potential of vemurafenib for improving long-term survival outcomes in HCC patients.

## Conclusion

Our study demonstrates that vemurafenib exhibits pronounced antitumor activity in HCC cells by suppressing proliferation, migration, and invasion, while promoting apoptosis and inducing cell cycle arrest in the G0/G1 phase. At the molecular level, vemurafenib activated JNK signaling and concurrently inhibited AKT phosphorylation, indicating that its anticancer efficacy is mediated through the modulation of key survival and apoptotic signaling cascades. Collectively, these results highlight the therapeutic potential of vemurafenib as a repurposed agent for HCC treatment, warranting further mechanistic and preclinical investigation.

## Abbreviations

HCC: Hepatocellular carcinoma
NSCLC: Non-small cell lung cancer
FDA: Food and Drug Administration
MAPK: Mitogen-activated protein kinase
JNK: c-Jun N-terminal kinase
AKT: Protein kinase B
PI3K: Phosphatidylinositol 3-kinase
mTOR: Mechanistic target of rapamycin
EMT: Epithelial-mesenchymal transition
Bcl-2: B-cell lymphoma 2
Bax: Bcl-2-associated X protein
CDK4: Cyclin-dependent kinase 4
p-JNK: Phosphorylated JNK
p-AKT: Phosphorylated AKT
FBS: Fetal bovine serum
PBS: Phosphate-buffered saline
CCK-8: Cell Counting Kit-8
LIHC: Liver hepatocellular carcinoma
GEO: Gene Expression Omnibus
ROS: Reactive oxygen species
ANOVA: Analysis of variance
SD: Standard deviation
